# Palmitoylethanolamide and Its Biobehavioral Correlates in Autism Spectrum Disorder: A Systematic Review of Human and Animal Evidence

**DOI:** 10.3390/nu13041346

**Published:** 2021-04-18

**Authors:** Marco Colizzi, Riccardo Bortoletto, Rosalia Costa, Leonardo Zoccante

**Affiliations:** 1 Child and Adolescent Neuropsychiatry Unit, Maternal-Child Integrated Care Department, Integrated University Hospital of Verona, 37126 Verona, Italy; riccardo.bortoletto91@gmail.com (R.B.); leonardo.zoccante@aovr.veneto.it (L.Z.); 2 Section of Psychiatry, Department of Neurosciences, Biomedicine and Movement Sciences, University of Verona, 37134 Verona, Italy; 3 Department of Psychosis Studies, Institute of Psychiatry, Psychology and Neuroscience, King’s College London, London SE5 8AF, UK; 4 Community Mental Health Team, Department of Mental Health, ASST Mantova, 46100 Mantua, Italy; rosalia.costa80@gmail.com

**Keywords:** neurodevelopment, pervasive developmental disorder, cannabinoids, acylethanolamines, immune response, glutamate, inflammation, peroxisome proliferator-activated receptor-α, child and adolescent neuropsychiatry

## Abstract

Autism spectrum disorder (ASD) pathophysiology is not completely understood; however, altered inflammatory response and glutamate signaling have been reported, leading to the investigation of molecules targeting the immune-glutamatergic system in ASD treatment. Palmitoylethanolamide (PEA) is a naturally occurring saturated N-acylethanolamine that has proven to be effective in controlling inflammation, depression, epilepsy, and pain, possibly through a neuroprotective role against glutamate toxicity. Here, we systematically reviewed all human and animal studies examining PEA and its biobehavioral correlates in ASD. Studies indicate altered serum/brain levels of PEA and other endocannabinoids (ECBs)/acylethanolamines (AEs) in ASD. Altered PEA signaling response to social exposure and altered expression/activity of enzymes responsible for the synthesis and catalysis of ECBs/AEs, as well as downregulation of the peroxisome proliferator activated receptor-α (PPAR-α) and cannabinoid receptor target GPR55 mRNA brain expression, have been reported. Stress and exposure to exogenous cannabinoids may modulate ECBs/AEs levels and expression of candidate genes for neuropsychiatric disorders, with implications for ASD. Limited research suggests that PEA supplementation reduces overall autism severity by improving language and social and nonsocial behaviors. Potential neurobiological underpinnings include modulation of immune response, neuroinflammation, neurotrophy, apoptosis, neurogenesis, neuroplasticity, neurodegeneration, mitochondrial function, and microbiota activity, possibly through peroxisome proliferator-activated receptor-α (PPAR-α) activation.

## 1. Introduction

Autism spectrum disorder (ASD) is a complex and multifactorial neurodevelopmental condition affecting those who suffer from it at different levels. Difficulties in social communication and associated tendency to have restrictive interests and repetitive and stereotyped activities are considered the core aspects of the disorder [1], allowing clinicians to make a diagnosis of ASD based on behavioral assessment [2]. However, converging research evidence supports the presence of altered neurobiological parameters such as functional and structural brain abnormalities [3,4], by which gene variants or epigenetic marks lead to the neurocognitive and biobehavioral symptoms consistently reported in ASD [5,6]. It is noteworthy that, even though the pathophysiology of ASD is not completely understood, studies conducted over the last two decades have helped clarify some of the mechanisms of disease progression. In particular, altered inflammatory response [7] and disrupted glutamate signaling [8,9,10] have been reported, leading the way to the investigation of the effect of molecules targeting the immune-glutamatergic system in improving clinical severity in ASD [11].

Growing evidence indicates that one of the major physiological functions of the cannabinoid signaling system is the modulation of neuroinflammation [12] and glutamate neurotransmission [13]. Both exogenous cannabinoids (e.g., plant-derived cannabinoids Δ9-tetrahydrocannabinol, Δ9-THC, and cannabidiol, CBD) and their endogenous counterparts (e.g., endocannabinoids anandamide and 2-arachidonoylglycerol, 2-AG) interact with the endocannabinoid system, with implications for health and disease [14,15]. Despite promising evidence on the efficacy of Δ9-THC- and CBD-based treatments in the management of ASD-related behavioral problems, it is still limited and requires further investigation [16]. Anandamide (arachidonoylethanolamide) and 2-AG are both derivatives of arachidonic acid, a polyunsaturated fatty acid well known as the precursor of bioactive prostaglandins and other eicosanoids, with anandamide specifically being its ethanolamine [17].

Other ethanolamines of various long-chain fatty acids also naturally occur, and along with anandamide they are collectively referred to as N-acylethanolamines. However, they are more abundant than anandamide in the body and do not bind to cannabinoid receptors, while exerting most of their biological effects by activating the peroxisome proliferator-activated receptor-α (PPAR-α), a nuclear receptor, and PPAR-α-independent pathways involving other receptors such as Transient Receptor Potential Vanilloid 1 (TRPV1) and GPR55 [17,18]. Research evidence suggests that such specific mechanisms of action would account for their anti-inflammatory, analgesic, anticonvulsant, and neuroprotective properties [17,18]. Palmitoylethanolamide (PEA, N-hexadecanoylethanolamide), in particular, is a saturated N-acylethanolamine which has been suggested to be effective in the control of inflammatory responses [19,20], depressive symptoms [21], epilepsy [22], and pain [23], possibly through a neuroprotective role against glutamate toxicity [17].

### Objectives

This effect of PEA on (neuro)inflammation and glutamate signaling could represent a promising neurobiological mechanism underlying the clinical utility of such N-acylethanolamine in ASD. This review aims to bring together and discuss all available data generated by clinical and preclinical studies investigating the role of PEA in ASD by conducting a systematic literature search for all such data. We reviewed all interventional and observational studies, employing either retrospective or prospective methodological approaches with any PEA biobehavioral correlates investigated in ASD.

## 2. Experimental Procedures

### 2.1. Inclusion and Exclusion Criteria

To summarize previous research evidence on the topic, inclusion criteria for studies were: (1) human or animal studies, (2) studies investigating both the acute and long-term effects of palmitoylethanolamide (PEA) administration in autism spectrum disorder (ASD) and related conditions, (3) studies investigating PEA signaling-related molecular markers in ASD and related conditions, including (a) blood serum levels, (b) brain tissue levels, (c) enzyme activity, and (d) receptors. Exclusion criteria were (1) studies where PEA was not the intervention of interest (i.e., studies evaluating only exogenous cannabinoid agonists or antagonists), (2) studies where PEA biobehavioral correlates were not investigated with reference to ASD, and (3) studies in which the PEA biobehavioral correlates were not directly reported on.

### 2.2. Search Strategy and Data Extraction

A literature search was conducted using electronic databases (MEDLINE, Web of Science and Scopus) for any published original study written in English, using a combination of search terms describing autism (‘autism spectrum disorder,’ ‘Asperger syndrome,’ ‘pervasive developmental disorder,’) and palmitoylethanolamide (‘palmitoylethanolamide,’ ‘palmitylethanolamide’, ‘N-(2-hydroxyethyl)hexadecanamide’, ‘N-(2-hydroxyethyl)palmitate’, ‘N-palmitoylethanolamine’) on 3 March 2021. Reference lists of eligible studies were also screened to identify additional eligible research. Publication data screening and extraction were performed following a 2-step selection process (conventional double-screening) conducted by 2 reviewers independently of each other (MC and RB).

### 2.3. Risk of Bias

The methodological heterogeneity of the studies (Table 1) included in this review necessitate a suitably inclusive and flexible approach to assess risk of bias and study quality. Thus, we used an adapted set of criteria suggested by the Agency for Healthcare Research and Quality (AHRQ) guidance [24], amended as appropriate for interventional and observational studies in humans (Table 2). Risk of systematic bias across human studies was additionally assessed by screening all papers for potential confounding variables such as comorbid psychiatric conditions and substance use (Table 2). Further, to assess any factor that may account for similarities and differences between animal studies, information was extracted about study characteristics, including animal model (e.g., mouse or rat), autism model (e.g., valproic acid, perinatal asphyxia, strain of idiopathic autism), developmental stage (i.e., prenatal or postnatal), sex, and PEA dosage (Table 3).

## 3. Results

### 3.1. Study Selection

**Figure 1:**
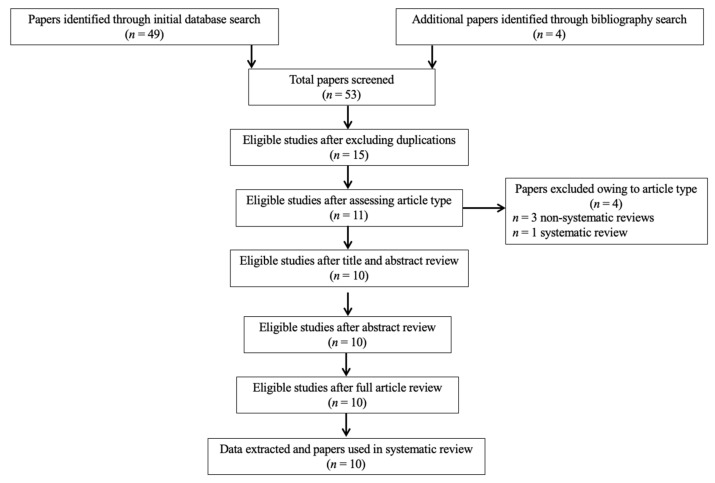
PRISMA flowchart of search strategy for systematic review.

In summary, 53 records were retrieved. Abstracts of all records were screened against the inclusion and exclusion criteria. After also excluding duplications (Figure 1), a final list of 10 studies (3 human, 6 animal, 1 using both humans and animals) was used for systematic analysis in this review (Table 1). In total, the eligible studies assessed different aspects of the palmitoylethanolamide (PEA) signaling pathway (Table 1). These include (1) in vivo PEA treatment exposure in human(s) with autism spectrum disorder by employing a monotherapy design (ASD; two studies; Table 2); (2) in vivo PEA treatment exposure in human(s) with ASD by employing an add-on design (one study; Table 2); (3) PEA quantitative blood assessment in human(s) with ASD (one study; Table 2); (4) in vivo PEA postnatal exposure in animal models of autism (two studies; Table 3); (5) in vivo PEA postnatal exposure in animal models of perinatal brain disorders (two studies; Table 3); (6) PEA quantitative brain assessment in animal models of autism (one study; Table 3); (7) PEA quantitative brain assessment in animal models of stress following exposure to exogenous cannabinoid agonists/antagonists (one study; Table 3); (8) PEA-related enzymes and receptors quantitative brain assessment in animal models of perinatal brain disorders (one study; Table 3). Additional data on methodological quality of studies conducted in humans and animals are reported in Table 2 and Table 3. A brief synthesis of the main results is presented below.

### 3.2. In Vivo PEA Treatment Exposure in Children and Adolescents with Autism Spectrum Disorder

Three studies have addressed this area in humans, including two case reports of two adolescents [25] and one child [26] with ASD as well as a double-blind, randomized (parallel), placebo-controlled trial performed among 62 risperidone-treated ASD children [27]. These studies have assessed the effects of PEA treatment on language [25,27], behavior [25,26,27] and additional parameters. The two case reports indicated that PEA supplementation improves expressive language [25] and reduces overall autism severity [25,26], including motor stereotypic behaviors [26], appearing to be beneficial also in modulating immune chemistry [25] and controlling enuresis [26]. The trial of PEA add-on to risperidone reported significant improvements in the domains of irritability and hyperactivity among ASD children receiving the combination treatment of risperidone and PEA as compared to those receiving only risperidone, with weak effects of PEA add-on also in reducing stereotypic behavior and inappropriate speech [27].

### 3.3. PEA Levels in Children and Adolescents with Autism Spectrum Disorder as Compared to Healthy Controls

This systematic review identified a single study specifically investigating whether ASD children and adolescents differ from their healthy peers in terms of circulating blood levels of PEA and other endocannabinoids (ECBs)/acylethanolamines (AEs). This study found that ASD children and adolescents have lower serum levels of PEA and other ECBs/AEs as compared to healthy controls. Additionally, PEA levels did not appear to change as a function of any sociodemographic and clinical characteristics [28].

### 3.4. In Vivo PEA Postnatal Exposure in Animal Models of Autism and Perinatal Brain Disorders

All these studies investigated the effect of PEA exposure, using similar but not overlapping methodologies in terms of animal type (mice [26,29], rat [30,31]), mode of administration (oral [26], subcutaneous [30,31], intraperitoneal [29]), period of exposure (postnatal day P15 to P120), dosage of PEA (1 to 30 mg/kg), and model of pathology (valproic acid (VPA) exposure [26], perinatal asphyxia (PA) [30,31], BTBR T+tf/J strain (BTBR) [29]). The first of these studies indicated that PEA administration improves social and nonsocial behaviors, reduces the expression of proinflammatory markers, modulates apoptosis in the hippocampus and cerebellum, and increases hippocampal neurogenesis and neuroplasticity in the VPA-induced rodent model of autism [26]. Another study found that PEA administration reverts the altered social and nonsocial behavioral phenotype in the inbred BTBR strain used as mice model of autism, possibly by reducing the expression of pro-inflammatory markers, activating the peroxisome proliferator-activated receptor-α (PPAR-α), and restoring hippocampal brain derived neurotrophic factor (BDNF) signaling, mitochondrial function, and microbiota composition [29]. Subsequent studies investigated the potential neuroprotective role of PEA in the PA rat model at P30 [30,31], since this postnatal age would correspond to the age of onset of neurodevelopmental conditions in humans. PEA treatment within the first hour of life attenuated the PA-induced alterations at P30 such as the altered prototypical behaviors [30], as well as the biochemical and morphological signs of altered neuronal cytoskeleton [30,31] and degeneration [30] in the hippocampus [30] and striatum [31].

### 3.5. Pea and Related Enzymes and Receptors Quantitative Brain Assessment in Animal Models of Autism, Perinatal Brain Disorders, and Stress Following Exposure to Exogenous Cannabinoids

In total, three studies did not evaluate the direct effect of PEA exposure while analyzing PEA levels and indirect measures of PEA signaling in the brain of animal models of autism and related disturbances [32,33,34]. A study found that alterations in various components of the endocannabinoid system could explain the autistic-like behavior observed among rats prenatally exposed to VPA, including increased hippocampal levels of PEA and other ECBs/AEs in response to social exposure, altered expression and activity of the enzymes responsible for the synthesis and catalysis of the 2-arachidonoylglycerol (2-AG) and reduced expression of PPAR-α and GPR55 mRNA across different brain areas [32]. Similarly, another study found that the behavioral alterations observed in the PA rat model could be due to a dysregulation of the enzymes responsible for the synthesis and catalysis of ECBs/AEs and a reduction in the expression of PPAR-α in the hippocampus [33]. Finally, a study found that both stress and exogenous manipulation of the endocannabinoid system affect ECBs/AEs levels and expression of candidate genes for neuropsychiatric disorders such as the cholinergic receptor nicotinic alpha 6 (Chrna6) and serotonin transporter neurotransmitter type 4 (Slc6a4) genes, with implications for the manifestation of aberrant behaviors [34].

## 4. Discussion

This is the first systematic review of all studies exploring the biobehavioral correlates of palmitoylethanolamide (PEA) in autism spectrum disorder (ASD) in humans and animals. Previous reviews have mainly addressed the potential role of neuroinflammation and altered glutamate signaling in ASD etiopathogenesis, indicating that, in subjects with genetic predispositions, atypical neurodevelopment may arise from a complex interplay between (neuro)inflammatory processes, mitochondrial dysfunction, oxidative stress and altered expression of glutamate signaling [35]. Interestingly, research evidence converges on the crucial role of exogenous cannabinoids and endocannabinoids in modulating such neurobiological systems, including neuroinflammation [12] and glutamate neurotransmission [13]. Overall, this review demonstrates that PEA may be involved in ASD, as indicated by interventional studies of the positive biobehavioral effects of PEA supplementation in both humans and animals as well as observational studies reporting aberrancies in the PEA signaling pathway at different levels.

PEA supplementation in humans as both monotherapy [25,26] and add-on therapy to antipsychotic medication [27] has been shown to reduce overall autism severity [25,26] by improving both expressive language (*what* the child says) [25] and inappropriate speech (*how* the child says it) [27], as well as modulating atypical behavior [25,26,27] and immune response [25]. Similarly, PEA supplementation in different animal models of autism and related conditions has been suggested to be effective in improving social and nonsocial behaviors [26,29,30] as well as in modulating a number of neurobiological processes including neuroinflammation [26], neurotrophy [29], apoptosis [26], neurogenesis [26], neuroplasticity [26], neurodegeneration [30,31], mitochondrial function [29], and microbiota activity [29]. Importantly, PEA administration resulted in an activation of the peroxisome proliferator activated receptor-α (PPAR-α) [29], whose downregulation may decrease antioxidative and anti-inflammatory processes, also altering energy homeostasis, mitochondrial fatty acid metabolism, and regulation of genes coding proteins that are involved in glutamate homeostasis and cholinergic/dopaminergic signaling in the brain, with implications for neurodevelopmental conditions [36]. The hippocampus is one of the brain areas where such modulatory effects of PEA are mostly reported. Further studies are needed to investigate the role of the PEA-induced modulation of the hippocampal system in improving the brain spatiotemporal framework within which sensory, emotional, and cognitive aspects of an experience are processed in ASD, with implications for comprehension and language production [37]. This is of paramount importance, as structural, functional, and neurochemical alterations in the hippocampus have been suggested as candidate biomarkers for the diagnosis of ASD in childhood [38].

Another line of research identified lower serum levels of PEA and other endocannabinoids (ECBs)/acylethanolamines (AEs) in humans suffering from ASD, independently of sociodemographic and clinical characteristics [28]. Similarly, studies performed in animal models of autism and related conditions suggested an altered response of the PEA signaling in the face of social exposure [32], altered expression and activity of the enzymes responsible for the synthesis and catalysis of ECBs/AEs [32,33], and downregulation of PPAR-α [32,33] and the cannabinoid receptor target GPR55 mRNA expression in the brain [32]. Interestingly, animals exposed to stress and administered with exogenous cannabinoids showed variations in their ECB/AE levels and expression of genes implied in neuropsychiatric disorders [34].

The findings of this systematic review have to be seen in light of some limitations. Research in the field is still too limited, especially in humans (2 of the 4 human studies are case reports, leaving a single experimental study conducted in a youth population with ASD), and further studies are needed to fully address the relevance of PEA for the different clinical phenotypes of ASD and whether the potentially beneficial effects of PEA are mediated by a protective role of the compound against altered neuroinflammatory responses and glutamate toxicity, which have been suggested to be involved in the pathophysiology of ASD [7,8,9,10]. Additionally, whether the PEA concentration profile can be used as a biomarker for ASD remains to be tested, and future longitudinal studies will have to investigate its clinical utility in monitoring response to treatment. Moreover, as the available evidence does not allow excluding a placebo effect [39], further clinical trials in larger samples are needed to fully explore the efficacy and tolerability of PEA supplementation in ASD. Finally, even though the systematic review followed the PRISMA statement, no review protocol was registered.

## 5. Conclusions

This review revealed a paucity of observational and experimental investigations of PEA and its pathway in ASD. However, the 10 studies discussed here seem to converge in reporting alterations of the PEA signaling, implications for ASD-related biobehavioral manifestations, and benefits from PEA supplementation. In particular, PEA may be useful in improving language difficulties and stereotypic behavior as well as in controlling hyperactivity and irritability which co-occur frequently in ASD. Notably, no serious adverse effects were observed with the administration of the compound in all human studies reviewed here, making PEA supplementation a potentially valid and reasonably safe therapeutic intervention in ASD.

## Figures and Tables

**Table 1:** Summary of studies investigating palmitoylethanolamide and its biobehavioral correlates in autism spectrum disorder.

Study (Country)	Aim of Study	PEA Type of Study	Population	N	Outcome Measure (Test Name or Description)	Results
Antonucci et al. (2015) (Italy)	To assess the effect of PEA on language, behavior, and immune chemistry	In vivo treatment exposure in humans	2 ASD adolescents	2	1. Expressive language (MLU); 2. overall autism severity (CARS-2; ATEC); 3. immune response (blood tests)	1. MLU: ↑ from 3.0 to 5.4; 2. CARS-2 test: ↓ from 43.5 to 32, ATEC: ↓ from 25 to 12; 3. vitamin D-OH25 level: ↑, CD57+ NK: ↑, total lymphocytes: ↓, total serum IgE: ↓ by ~50% (only in 1 case, in the other case NS), atopic illnesses: ↓, total WC: unchanged
Aran et al. (2019) (Israel)	To assess PEA and other ECBs/AEs blood levels and their association with behavior	Quantitative blood assessment in humans	1. 93 ASD children/adolescents; 2. 93 HCs	186	Serum ECBs/AEs levels (LC-MS/MS)	1. **Serum levels of AEA, OEA and PEA: ASD < HC (after Bonferroni correction, still significant)**, 2-AG: ASD vs. HC, NS; 2. **↓ age or ↓ BMI: ↓serum levels of AEA** (after Bonferroni correction, NS; correlations with other ECBs/AEs, NS); 3. **↑ APSI severity score or ↑ use of antipsychotics: ↓ serum levels of AEA (trend effect**; correlations with other ECBs/AEs and/or other symptoms, NS)
Bertolino et al. (2016) (Italy)	To assess the effect of PEA on behavior	In vivo treatment exposure in humans	1 ASD child	1	1. Overall autism severity (ATEC); 2. Motor stereotypic behaviors (Quarterly questionnaire)	1. ATEC: ↓ of both total and subgroup scores (Speech, Sociability, Sensory/Cognitive, Health/Physical/Behavior; improved of about 23%); 2. motor stereotypies: ↓; enuresis: ↓ from 91% to 2.4% after 14 months
Khalaj et al. (2018) (Iran)	To assess the effect of PEA add-on to risperidone on language and behavior	In vivo treatment exposure in humans	1. 31 ASD (Arm A, risperidone + PEA); 2. 31 ASD children (Arm B, risperidone + placebo)	62 (out of 70 randomized)	Behavior (ABC-C)	1. **ABC-C over time: irritability and hyperactivity, risperidone + PEA < risperidone + placebo** (other domains NS); 2. **ABC-C at 10 weeks: irritability, hyperactivity, and inappropriate speech (trend), risperidone + PEA < risperidone + placebo** (other domains NS); 3. **ABC-C at 5 weeks: hyperactivity, stereotypic behavior (trend), and inappropriate speech (trend), risperidone + PEA < risperidone + placebo** (other domains NS); 4. **ABC-C response rate at 10 weeks, hyperactivity, irritability, and inappropriate speech, risperidone + PEA > risperidone + placebo** (other domains NS); 5. ABC-C response rate at 5 weeks, NS; Adverse events, NS
Bertolino et al. (2016) (Italy)	To assess the effect of PEA on behavior, neuroinflammation, neuromodulation, and neurogenesis	In vivo postnatal exposure in animals	1. 60 SHAM+VHI; 2. 60 SHAM+PEA; 3. 60 VPA+VHI; 60 VPA+PEA	240	1. Behavior (SIT; EPM); 2. Neuroinflammation, neuromodulation, and neurogenesis (Immunohistochemistry (Chymase, Tryptase, TNF-α, IL-1β); Western Blot (Bax, Bcl-2, iNOS, IкBα, NF-kB, GFAP); Neurogenesis (BrdU and DCX Immunohistochemistry and Golgi impregnation))	1. **SIT: stay duration in stranger side, VPA < VPA+PEA; stay duration in central area, VPA > VPA+PEA**; **SI, VPA < VPA+PEA; EPM: time spent in the open arm, VPA < VPA+PEA; 2. chymase, tryptase, IL-1 and TNF-α expression to mast cells in hippocampus and cerebellum: VPA > VPA+PEA; iNOS and GFAP levels in hippocampus and cerebellum: VPA > VPA+PEA; IкBα and NF-kB levels: VPA < VPA+PEA; Bax: VPA > VPA+PEA; Bcl-2: VPA < VPA+PEA; BrdU+ cells, DCX+ cells and development of dendritic spines in the dentate gyrus of hippocampus: VPA < VPA+PEA**
Kerr et al. (2013) (Ireland)	To assess PEA and other ECBs/AEs brain levels and their association with behavior, and ECBs/AEs-related gene expression and enzyme activity	Quantitative brain assessment in animals	1. 16 saline; 2. 14 VPA	30	1. Brain ECBs/AEs levels (LC-MS/MS); 2. behavior (sociability test); 3. gene expression (real-time PCR); 4. enzyme activity	1. AEA, 2-AG, PEA and OEA in frontal cortex, hippocampus and cerebellum: VPA vs. saline NS; 2. **AEA, OEA and PEA in hippocampus after sociability test: VPA > saline** (other ECBs in other brain regions NS); 3. **MAGL mRNA in the hippocampus, DAGLα mRNA in the cerebellum, VPA < saline** (other brain region NS); **PPARα and PPARγ mRNA in frontal cortex and hippocampus, VPA < saline**; **GPR55 mRNA in frontal cortex and hippocampus, VPA < saline** (cerebellum NS); CB_1_/CB_2_ receptor mRNA in frontal cortex, hippocampus and cerebellum, VPA vs. saline, NS; 4. **MAGL activity in the hippocampus, VPA > saline** (FAAH NS)
Blanco et al. (2015) (Spain)	To assess ECBs/AEs-related enzyme and receptor activity	Quantitative brain assessment in animals	1.CTL; 2. C+; 3. PA	15 (mothers)	1. Brain ECBs/AEs-related enzyme and receptor activity (immunohistochemistry, immunostaining quantification)	1. NeuN: NS; **GFAP-positive cells: PA > CTL and C+ in CA1, CA3, and DG regions of dorsal hippocampus**; **DAGLα expression: PA and C+ > CTL in CA1, C+ > CTL in DG** (other regions or groups NS); **NAPE-PLD expression: PA < C+ and CTL in CA1 and CA3, PA < C+ in DG** (other regions or groups NS); CB1 expression, NS; **PPARα-positive cells: PA < C+ and CTL in CA1 and CA3** (DG NS); **FAAH expression: C+ > CTL and PA in CA3** (other regions or groups NS)
Herrera et al. (2018) (Spain)	To assess the effect of PEA on behavior and neuronal damage	In vivo postnatal exposure in animals	1. 15 PA+VHI; 2. 13 CTL (VHI); 3. 18 PA+PEA; 4. 17 CTL+PEA	63	1. Behavior (OFT, EPM); 2. neuronal damage (electron microscopy, immunohistochemistry (NeuN, pNF-H/M, MAP-2, GFAP), Western Blot (pNF H/M, MAP-2, GFAP))	1. **OFT: time spent rearing, PA < CTL, PA < PA+PEA,** CTL vs. PA+PEA NS, CTL vs. CTL+PEA NS; **time spent grooming, PA > CTL, PA > PA+PEA,** CTL vs. PA+PEA NS, CTL vs. CTL+PEA NS; **EPM: time spent rearing, PA < CTL, PA < PA+PEA,** CTL vs. PA+PEA NS, CTL vs. CTL+PEA NS; **time spent grooming, PA > CTL**, PA vs. PA+PEA NS, **PA+PEA > CTL**; **time spent HD, PA > CTL, PA > PA+PEA**, CTL vs. PA+PEA NS, CTL vs. CTL+PEA NS; 2. **pyknotic nucleus in the hippocampal CA1 neurons: PA > CTL, ↓ in PA+PEA; NeuN abnormal neurons in the hippocampal CA1 layer: PA > CTL, ↓ in PA+PEA; hippocampal pNF-H/M reactive area/ protein expression: PA > CTL, ↓in PA+PEA** (PA+PEA still > CTL); **hippocampal MAP-2reactive area/ protein expression: PA < CTL, ↑ in PA+PEA** (PA+PEA still < CTL); GFAP-positive cells and protein expression: NS
Cristiano et al. (2018) (Italy)	To assess the effect of PEA on behavior, gene expression, receptor activity, neurotrophins, mitochondrial function, neuroinflammation, and microbiota-gut-brain axis	In vivo postnatal exposure in animals	1. C57Bl/6J+VHI (control B6); 2. BTBR T+tf/J+VHI (BTBR); 3. BTBR+PEA; 4. BTBR+GW; 5. BTBR+GW+PEA; 6. B6 PPAR-α null (KO)+VHI; 7. KO+PEA	6–12 per group of experiment	1. Behavior (MBA, SGT, TST); 2. gene expression, receptor activity, neurotrophins (Western Blot, real-time PCR); 3. mitochondrial function and neuroinflammation (serum parameters, mitochondrial parameters, oxydative stress assay); 4. microbiota-gut-brain axis (intestinal permeability assay (FITC-labeled dextran, faecal microbiota))	1. **MBA and SGT**: **BTBR > B6, BTBR+PEA10 ↓, BTBR+PEA30 ↓↓**, BTBR+GW+PEA vs. BTBR NS, BTBR+GW vs. BTBR NS, KO+PEA vs. KO NS; **TST: BTBR+PEA10 ↑, BTBR+PEA30 ↑↑**, BTBR+GW+PEA vs. BTBR NS, BTBR+GW vs. BTBR NS, KO+PEA vs. KO NS; BTBR+PEA30 early (1h) effect NS; B6+PEA vs. B6 NS; 2. **PPAR-α mRNA and protein, BDNF protein, TrkB mRNA, CREB protein** (CREB mRNA NS): **BTBR < B6, BTBR+PEA30 ↑**; 3. **mitochondrial state 3 respiration and SOD activity: BTBR < B6, BTBR+PEA ↑** (mitochondrial state 4 respiration and oligomycin state 4 respiration NS); **FCCP-stimulated respiration, mitochondrial energetic efficiency, ROS: BTBR+PEA ↓**; **TNFα, IL1-β, IL6 in hippocampus, colon and serum: BTBR > B6, BTBR+PEA ↓**; 4. **epithelial barrier integrity (Tjp1 and Ocln mRNA levels): BTBR < B6, BTBR+PEA ↑; Microbial community: Firmicutes/Bacteroidetes ratio, BTBR+PEA > BTBR**
Tomas-Roig et al. (2018) (Germany)	To assess the effect of repeated stress and acute cannabinoid exposure on behavior and ECBs/AEs brain levels	Quantitative brain assessment in animals	1. 60 STS; 2. 60 CTL; 3. VHI; 4. WIN+VHI; 5. Rim+VHI; 6. Rim+WIN; 7.STS+VHI; 8. STS+WIN+VHI; 9. STS+Rim+VHI; 10. STS+Rim+WIN; 11. CTL+VHI; 12. CTL+WIN+VHI; 13. CTL+Rim+VHI; 14. CTL+Rim+WIN	120	1. Behavior (FOB, OFT); 2. brain ECBs/AEs and related gene expression (LC-APCI-MS, real-time PCR)	1. **Effect of stress: AEA levels in dorsal CPu, STS < CTL; Chrna6 gene expression, STS > CTL**; 2. **Effect of drugs exposure: OFT, distance travelled, WIN+VHI < VHI, Rim+WIN < Rim+VHI; distance travelled in center, Rim+VHI > WIN+VHI; rearing activity, Rim+WIN > WIN+VHI; PEA and OEA levels in dorsal Cpu, WIN+VHI > others; Fkpb5 expression, Rim+WIN > others**; 3. **Effect of stress under drug influence: OFT, distance travelled, STS+VHI > CTL+VHI, STS/CTL+WIN+VHI < STS/CTL+Rim+VHI, STS+WIN+VHI < STS+VHI, CTL+Rim+VHI > CTL+VHI; 2-AG levels, CTL+Rim+VHI > CTL+VHI/CTL+Rim+WIN; Chrna6 gene expression: CTL+WIN+VHI/CTL+Rim+VHI/CTL+Rim+WIN > CTL+VHI, STS+Rim+VHI/STS+Rim+WIN > STS+WIN+VHI/CTL groups; Slc6a4 gene expression: Rim+VHI > others**
Udovin et al. (2020) (Argentina)	To assess the effect of PEA on neuronal damage	In vivo postnatal exposure in animals	1. 15 PA+VHI; 2. 13 CTL (VHI); 3. 18 PA+PEA; 4. 7 CTL+PEA	53	1. Neuronal damage (electron microscopy, immunohistochemistry (pNF-H/M, MAP-2, GFAP), Western Blot (pNF H/M, MAP-2, GFAP, antiglyceraldehyde-3-phosphate dehydrogenase))	1. **Striatal pNF-H/M reactive area/protein expression: PA < CTL, ↑ in PA+PEA** (PA+PEA still < CTL); **striatal MAP-2 reactive area/protein expression: PA < CTL, ↑ in PA+PEA**; **GFAP-positive cells: PA < CTL, ↑ in PA+PEA** (PA+PEA still < CTL; protein expression NS)

PEA, Palmitoylethanolamide; ASD, Autism Spectrum Disorder; MLU, Mean Length of Utterance; CARS-2, Childhood Autism Rating Scale-Second Edition; ATEC, Autism Treatment Evaluation Checklist; 25(OH)D3, 25-hydroxyvitamin D3; NK, Natural Killer cells; IgE, Immunoglobulin E; NS, Not Significant; WCs, White Cells; ECBs, Endocannabinoids; HCs, Healthy Controls; LC–MS/MS, Liquid Chromatography–Mass Spectrometry; AEA, Arachidonoylethanolamide; OEA, Oleoylethanolamide; 2-AG, 2-arachidonoyglycerol; BMI, Body Mass Index; APSI, Autism Parenting Stress Index; ABC-C, Aberrant Behavior Checklist-Community; VPA, Valproate; SIT, Social Interaction Test; EPM, Elevated Plus Maze; TNF-α, Tumor Necrosis Factor alpha; IL-1β, Interleukin 1 beta; Bax, BCL2-Associated X protein; Bcl-2, B-cell lymphoma 2; iNOS, inducible Nitric Oxide Synthase; IкBα, nuclear factor of kappa light polypeptide gene enhancer in B-cells inhibitor, alpha; NF-kB, nuclear factor kappa-light-chain-enhancer of activated B cells; GFAP, Glial Fibrillary Acidic Protein; BrdU, Bromodeoxyuridine; DCX, Doublecortin; AEs, acylethanolamines; PCR, Polymerase Chain Reaction; MAGL, Monoacylglycerol lipase; mRNA, Messenger Ribonucleic Acid; DAGLα, Diacylglycerol lipase α; PPARα, Peroxisome Proliferator-Activated Receptor alpha; PPARγ, Peroxisome Proliferator-Activated Receptor gamma; GPR55, G protein-coupled receptor 55; CB1, Cannabinoid receptor type 1; CB2, Cannabinoid receptor type 2; FAAH, Fatty Acid Amide Hydrolase; CTL, control group; C+, cesarean section group; PA, Perinatal Asphyxia; CA1, Cornu Ammonis-1; CA3, Cornu Ammonis-3; DG, Dentate Gyrus; NAPE-PLD, N-acyl phosphatidylethanolamine phospholipase D; OFT, Open Field Test; NeuN, Hexaribonucleotide Binding Protein-3; pNF-H/M, Phosphorylated Neurofilament H; MAP-2, Microtubule-associated protein 2; HD, Head dipping; GW, GW6471 (N-((2S)-2-(((1Z)-1-Methyl-3-oxo-3-(4-(trifluoromethyl)phenyl)prop-1-enyl) amino)-3-(4-(2-(5-methyl-2-phenyl-1,3-oxazol-4-yl)ethoxy)phenyl)propyl) propanamide); MBA, Marble Burying Assay; SGT, Self-Grooming Test; TST, Three-chambered Social Test; FITC, Fluorescein Isothiocyanate; PEA10, Palmitoylethanolamide 10 mg/Kg; PEA30, Palmitoylethanolamide 30 mg/Kg; 1h, one hour; BDNF, Brain-Derived Neurotrophic Factor; TrkB, Tropomyosin receptor kinase B; CREB, cAMP response element-binding protein; SOD, Superoxide Dismutase; FCCP, Carbonyl cyanide-4 (trifluoromethoxy) phenylhydrazone; ROS, Reactive Oxygen Species; IL6, Interleukin 6; Tjp1, Tight junction protein 1; Ocln, Occludin; STS, stress; WIN, CB1/CB2 receptor agonist WIN55212.2; Rim, selective cannabinoid CB1 receptor antagonist Rimonabant; FOB, Functional Observational Battery; LC–APCI–MS, liquid chromatography–atmospheric pressure chemical ionization–mass spectrometry; Cpu, caudate-putamen; Chrna6, Cholinergic Receptor Nicotinic Alpha 6; Fkpb5, FKBP Prolyl Isomerase 5; Slc6a4, Solute Carrier Family 6 Member 4; bold font emphasizes statistically significant results.

**Table 2:** Methodological quality of human studies investigating palmitoylethanolamide and its biobehavioral correlates in autism spectrum disorder.

Study	Study Design	Defined Study Population	Age (Years)	Gender	PEA Measure	Adequate PEA Evaluation	Control	Comparability of Subjects	Other Comorbidity	Excluded/Adjusted for Confounding Factors	Statistical Analyses	Funding or Sponsorship
Antonucci et al. (2015) (Italy)	√ Case report	√ Clinical diagnosis	√ 1. 13 years old; 2. 15 years old	√ Male	√ 1. Normast 600 mg, 1/2 tablet twice daily for one week, THEN 1 tablet twice daily (oral administration); 2. Normast 600 mg, once daily (oral administration)	√ 1. 1 month exposure; 2. 3 month exposure	X	NA	√ 1. Atopia; 2. Epilepsy	X	NA	√
Aran et al. (2019) (Israel)	√ Analytic, observational	√ ADOS-2, DSM-5, additional assessments (VABS-II, SCQ lifetime form, CARS2-ST, HSQ-ASD, CBCL-validated Hebrew version, APSI, SRS-2 Hebrew version, CGI-S)	√ Mean (SD) [range]: 1. HC: 11.8 (4.3) [5.5-21]; 2. ASD: 13.1 (4.1) [6–21]	√ Male (%): 1. HC: 79%; 2. ASD: 79%	√ Serum blood levels	√ Single assessment	√	√ Matched for age, gender and BMI	√ 1. HC, no neuropsychiatric comorbidity other than ADHD	√ 1. Results adjusted for age, gender, BMI, and ADHD; 2. results correlated with anxiety, behavior, epilepsy, and perinatal complication comorbidity	√ t-test, Bonferroni correction, Pearson χ2 test, Pearson correlation, multivariate logistic regression, linear regression	√
Bertolino et al. (2016) (Italy)	√ Case report	√ Clinical diagnosis	√ 10 years old	√ Male	√ co-ultraPEA-Lut 700 mg + 70 mg twice daily (oral administration)	√ 12/14 months	X	NA	√ Tetralogy of Fallot	X	NA	√
Khalaj et al. (2018) (Iran)	√ Double-blind, randomized (parallel), placebo controlled	√ DSM-5, having irritability symptoms of at least moderate severity (scores ≥ 12 on the ABC-C Irritability subscale).	√ Mean (SD) [range]: 1. Risperidone + PEA, 6.84 (2.1) [4–12]; 2. Risperidone + placebo, 7.42 (2.35) [4–12]	√ Male, N (%): 1. Risperidone + PEA, 22 (70.97); 2. Risperidone + placebo, 25 (80.65)	√ 600 mg twice daily (oral administration)	√ 10 weeks	√	√ Matched for age, sex, weight, ESRS score, ABC-C irritability, lethargy, stereotypy, hyperactivity, and inappropriate speech domains	√ Exclusion criterion	√ Excluded if 1. symptoms not so severe for treatment with risperidone; 2. concomitant psychiatric disorder; 3. preexisting medical condition; 4. severe intellectual disability; 5. alcohol/drug abuse; 6. dyskinesia; 7. antipsychotic medication or behavior treatment within the past 6 months	√ t-test with Levene’s test for equality of variance, Freeman-Halton extension of Fisher’s exact test, Cohen’s d, repeated measures ANOVA, ITT	√

Mg, milligrams; NA, not applicable; ADOS-2, Autism Diagnostic Observatory Schedule Second Edition; DSM-5, Diagnostic and Statistical Manual of Mental Disorders fifth edition; VABS-II, Vineland Adaptive Behavior Scale Second Edition; SCQ, Social Communication Questionnaire; CARS2-ST, Childhood Autism Rating Scale-second edition; HSQ-ASD, Home Situations Questionnaire-Autism Spectrum Disorder; CBCL, Child Behavior Checklist; APSI, Autism Parenting Stress Index; SRS-2, Social Responsiveness Scale-II; CGI-S, Clinical Global Impression-Severity; SD, standard deviation; HCs, healthy controls; ASD, Autism Spectrum Disorder; BMI, Body Mass Index; ADHD, Attention Deficit Hyperactivity Disorder; co-ultraPEA-Lut, ultramicronized Palmitoylethanolamide with Luteolin; ABC-C, Aberrant Behavior Checklist Community; PEA, Palmitoylethanolamide; ESRS, Extrapyramidal Symptom Rating Scale; ANOVA, Analysis of Variance; ITT, intention-to-treat.

**Table 3:** Methodological quality of animal studies investigating palmitoylethanolamide and its biobehavioral correlates in autism spectrum disorder.

Study	Study Design	Defined Study Population	Age	Gender	PEA Measure	Adequate PEA Evaluation	Control Group	Statistical Analyses	Funding or Sponsorship
Bertolino et al. (2016) (Italy)	√ Analytic, observational, interventional	√ C57/BL6 mice injected SC with VPA (400 mg/kg) on P14	√ P15-P120	√ Male	√ co-ultraPEA-LUT 1 mg/kg (oral administration by gavage)	√ 1. 2 weeks for behavior, immunochemistry and Western Blot studies; 2. 3 months for neurogenesis studies	√ SHAM/VPA + vehicle, SHAM+PEA	√ Behavior: one-way ANOVA, Newman–Keuls multiple comparison test; Immunohistochemistry: ANOVA and post hoc Tukey tests with Bonferroni correction for multiple comparisons; all other results: ANOVA and Bonferroni post hoc for multiple comparisons	√
Kerr et al. (2013) (Ireland)	√ Analytic, observational	√ Litters of female Sprague Dawley rats SC injected with VPA (600 mg/kg) at G12.5	√ P33-P40	√ Male and female	√ Brain tissue levels	√ Single assessment	√ Saline-treated	√ Shapiro–Wilk test; Levene test; unpaired t-test	√
Blanco et al. (2015) (Spain)	√ Analytic, observational	√ Rats exposed to PA procedures	√ P30	√ Male	√ Brain tissue levels of PEA-related enzymes and receptors	√ Single assessment	√ CTL, C+	√ ANOVA, Tukey’s post hoc tests for multiple comparisons, Bonferroni’s correction, Kuskal-Wallis test, Mann–Whitney test	√
Herrera et al. (2018) (Spain)	√ Analytic, observational, interventional	√ Rats exposed to PA procedures	√ P30	√ Male	√ 10 mg/Kg (SC injection)	√ Single administration (within the 1st h of life)	√ CTL/PA + vehicle, CTL+PEA	√ Shapiro–Wilk test; Levene test; ANOVA; Student’s t-test; Bonferroni’s correction	√
Cristiano et al. (2018) (Italy)	√ Analytic, observational, interventional	√ BTBR T+tf/J (BTBR) mice	√ 3–4 months	√ Male	√ 10 or 30 mg/Kg (IP injection)	√ Daily administration (10 days)	√ B6/BTBR/BTBR+GW/KO + vehicle, BTBR+GW/KO + PEA	√ ANOVA, Bonferroni’s correction	√
Tomas-Roig et al. (2018) (Germany)	√ Analytic, observational, interventional	√ C57BI6/J mice exposed to stress (1 h/day per 21 days)	√ 7-8 weeks	√ Male	√ Brain tissue levels	√ Single assessment	√ CTL (left undisturbed)	√ ANOVA, Brown–Forsythe test, Bonferroni’s correction, Tamhane post hoc test, Student’s t-test	√
Udovin et al. (2020) (Argentina)	√ Analytic, observational, interventional	√ Rats exposed to PA procedures	√ P30	√ Male	√ 10 mg/Kg (SC injection)	√ Single administration (within the 1st h of life)	√ CTL/PA + vehicle, CTL+PEA	√ Shapiro–Wilk test; Levene test; ANOVA; Student’s t-test; Bonferroni’s correction	√

VPA, Valproate; mg/kg, milligrams per kilogram; SC, subcutaneously; P, postnatal day 14; P15, postnatal day 15; P120, postnatal day 120; co-ultraPEA-Lut, ultramicronized Palmitoylethanolamide with Luteolin; PEA, Palmitoylethanolamide; ANOVA, Analysis of Variance; G12.5, gestational day 12.5; P33, postnatal day 33; P40, postnatal day 40; PA, Perinatal Asphyxia; P30, postnatal day 30; CTL, control group; C+, cesarean section group; 1st h, first hour; IP, intraperitoneal; GW, GW6471 (N-((2S)-2-(((1Z)-1-Methyl-3-oxo-3-(4-(trifluoromethyl)phenyl)prop-1-enyl) amino)-3-(4-(2-(5-methyl-2-phenyl-1,3-oxazol-4-yl)ethoxy)phenyl)propyl) propanamide); h/day, hour per day.

## Data Availability

Not applicable.
